# Comparative Genomics Unravels the Functional Roles of Co-occurring Acidophilic Bacteria in Bioleaching Heaps

**DOI:** 10.3389/fmicb.2017.00790

**Published:** 2017-05-05

**Authors:** Xian Zhang, Xueduan Liu, Yili Liang, Yunhua Xiao, Liyuan Ma, Xue Guo, Bo Miao, Hongwei Liu, Deliang Peng, Wenkun Huang, Huaqun Yin

**Affiliations:** ^1^School of Minerals Processing and Bioengineering, Central South UniversityChangsha, China; ^2^Key Laboratory of Biometallurgy of Ministry of Education, Central South UniversityChangsha, China; ^3^State Key Laboratory for Biology of Plant Diseases and Insect Pests, Institute of Plant Protection, Chinese Academy of Agricultural SciencesBeijing, China

**Keywords:** bioleaching heaps, co-occurring bacteria, comparative genomics, functional roles, mutualistic interaction

## Abstract

The spatial-temporal distribution of populations in various econiches is thought to be potentially related to individual differences in the utilization of nutrients or other resources, but their functional roles in the microbial communities remain elusive. We compared differentiation in gene repertoire and metabolic profiles, with a focus on the potential functional traits of three commonly recognized members (*Acidithiobacillus caldus, Leptospirillum ferriphilum*, and *Sulfobacillus thermosulfidooxidans*) in bioleaching heaps. Comparative genomics revealed that intra-species divergence might be driven by horizontal gene transfer. These co-occurring bacteria shared a few homologous genes, which significantly suggested the genomic differences between these organisms. Notably, relatively more genes assigned to the Clusters of Orthologous Groups category [G] (carbohydrate transport and metabolism) were identified in *Sulfobacillus thermosulfidooxidans* compared to the two other species, which probably indicated their mixotrophic capabilities that assimilate both organic and inorganic forms of carbon. Further inspection revealed distinctive metabolic capabilities involving carbon assimilation, nitrogen uptake, and iron-sulfur cycling, providing robust evidence for functional differences with respect to nutrient utilization. Therefore, we proposed that the mutual compensation of functionalities among these co-occurring organisms might provide a selective advantage for efficiently utilizing the limited resources in their habitats. Furthermore, it might be favorable to chemoautotrophs' lifestyles to form mutualistic interactions with these heterotrophic and/or mixotrophic acidophiles, whereby the latter could degrade organic compounds to effectively detoxify the environments. Collectively, the findings shed light on the genetic traits and potential metabolic activities of these organisms, and enable us to make some inferences about genomic and functional differences that might allow them to co-exist.

## Introduction

Unraveling the ecological and functional roles of microorganisms in biological communities is an important but still elusive issue (Prosser et al., 2007), even though these microbes are thought to be crucial to the function ecosystems (Harris, 2009; Jiao et al., 2010; Hua et al., 2015). As stated by Sogin et al. (2006), there is a surprisingly wide biodiversity of microbial communities in pristine environments. In their study, the dominate populations are numerically significant, but the members of the rare biosphere account for the majority of the phylogenetic diversity. Similar results were generally observed in other natural and anthropogenic environments based on metagenomic and metatranscriptomic analyses (Chen et al., 2015; Goltsman et al., 2015; Xiao et al., 2016; Zhang et al., 2016d). Genomes of microbial members in various communities have been reconstructed with the benefit of cultivation-independent sequencing (Tyson et al., 2004; Mason et al., 2012; Wu et al., 2016), providing a first glimpse of their functional roles *in situ*. Additionally, several bioinformatics-based strategies have been attempted to obtain genomic assemblies from metagenomic datasets (Dick et al., 2009; Hua et al., 2015). Considerable efforts have been made to expand the scope of microbial genetics and ecophysiology on a global scale; however, relatively little is known about how these populations co-exist within the same microbial community.

Acidophilic microorganisms are widely distributed in both pristine environments (e.g., acid rock drainage and volcanic, geothermal areas) and acidic environments of anthropogenic origin (e.g., acid mine drainage and bioleaching heaps, Baker and Banfield, 2003; Denef et al., 2010; Bonnefoy and Holmes, 2012; Zhang et al., 2016c,d). Many acidophiles that are metabolically active in anthropogenic environments are obligate chemolithoautotrophs capable of assimilating atmospheric CO_2_ and deriving energy from the aerobic oxidation of ferrous iron and/or a variety of sulfur species (Hallberg and Johnson, 2001; Rawlings, 2005; Johnson and Hallberg, 2008). In addition, heterotrophs that assimilate organic carbon and mixotrophs that utilize both organic and inorganic forms of carbon are found in these acidic settings (Johnson and Hallberg, 2003).

Until recently, numerous pieces of evidence suggested that *Acidithiobacillus* and *Leptospirillum* are considered to be the common inhabitants of acidophilic, metal-tolerant microbial consortia in many sulfide-rich mining environments (Kock and Schippers, 2006, 2008; Breuker et al., 2009; Chen et al., 2015; Xiao et al., 2016; Zhang et al., 2016d). Species of *Acidithiobacillus* isolates are demonstrated iron- and/or sulfur-oxidizing acidophiles that are phylogenetically affiliated with the class *Acidithiobacillia* (Williams and Kelly, 2013), which mainly includes *A. ferrooxidans* (Valdés et al., 2008), *A. thiooxidans* (Yin et al., 2014), *A. caldus* (Valdes et al., 2009), and *A. ferrivorans* (Liljeqvist et al., 2011). Four recognized members of iron-oxidizing *Leptospirillum* bacteria (Zhang et al., 2016c), including Group I (*L. ferrooxidans*, Fujimura et al., 2012), Group II (*L. rubarum* and *L. ferriphilum*, Jiang et al., 2015), Group III (*L. ferrodiazotrophum*), and Group IV (Goltsman et al., 2013), have been documented. In contrast to these obligate chemolithoautotrophs, heterotrophic and/or mixotrophic acidophiles, such as *Sulfobacillus* spp., have been also characterized from these environments (Guo et al., 2014; Justice et al., 2014). Despite relatively poor understanding of the moderately thermophilic *Sulfobacillus*, it was believed to play an important role in the biogeochemical cycle of sulfur (Justice et al., 2014). Several isolated *Sulfobacillus* species, including *S. benefaciens* (Johnson et al., 2008), *S. thermosulfidooxidans* (Justice et al., 2014), *S. sibiricus* (Melamud et al., 2003), and *S. thermotolerans* (Bogdanova et al., 2006), have been used to demonstrate their key metabolic features.

Interactions occur ubiquitously among co-existing microbes in laboratory cultures and natural environments (Li and Gu, 2007; Summers et al., 2010; Wintermute and Silver, 2010; Gupta and Schuster, 2013; Yin et al., 2015). Recently, a striking example of an inter-species interaction was found between *Arthrobacter* sp. and *Sphingopyxis* sp. in a co-culture (Liang et al., 2017) where Mn(II)-oxidizing activity was presumed to be triggered by contact-dependent interactions of two investigated bacteria, although neither of them has the ability to oxidize Mn(II). As in other environments, microbial interactions in mixed communities of acidic environments were also described (Johnson, 1998; Baker and Banfield, 2003). The spatial-temporal distribution of different populations might be correlated with the differences in environmental conditions of individual ecological niches, and in nutrients or in other resources that they could utilize (Yelton et al., 2013). Thus, it is important to investigate how these physiologically different acidophilic microorganisms interact, thereby allowing them to co-exist.

Here, we present the detailed analyses of gene repertoire, metabolic features, and potential functional roles of three acidophilic species, including *Acidithiobacillus caldus, Sulfobacillus thermosulfidooxidans*, and *Leptospirillum ferriphilum*, which were isolated from disparate bioleaching heaps located in Dexing Copper Mine (Jiangxi Province, China) and Zijinshan Copper Mine (Fujian Province, China), respectively. A comparative survey based on the bacterial genomes was performed to delineate the genomic and functional differences among these co-occurring acidophiles that potentially contribute to a mutualistic relationship rather than competitive exclusion.

## Materials and methods

### Sampling, DNA extraction, genome sequencing, and assembly

Samples were collected from mine tailings heaps in Dexing Copper Mine (Jiangxi, China) and Zijinshan Copper Mine (Fujian, China). Leaching solution that harbored plentiful microorganisms was pumped from leaching pools and sprayed on the leaching heaps periodically in the process of industrial bioleaching operations. Environmental attributes of these two sampling sites have been elaborated on in earlier studies (Yin et al., 2008; Xiao et al., 2016). All bioleaching samples were repeatedly washed with distilled water (pH 2.0), and then they were filtered through the filter membrane with a 0.22-μm pore-size as described earlier (Zhang et al., 2016d). Gradient dilution was employed to isolate the pure bacteria from these environmental samples according to individual growth conditions of targeted isolates. In general, all of the strains were grown in liquid 9K medium on a shaking table at 170 rpm. Additional details are as follows: autoclave-sterilized elemental sulfur (10 g/L), 45°C, and pH 2.0 for *Acidithiobacillus caldus*; and 50 mM of ferrous [Fe(II)], 40°C, and pH 1.5 for *Leptospirillum ferriphilum*. The culture conditions of *S. thermosulfidooxidans* were previously documented (Zhang et al., 2017).

Strains were cultivated aerobically at specific conditions as above. Bacterial cells were harvested at the stationary phase by centrifugation (12,000 g) for 10 min at 4°C. Genomic DNA was extracted using a TIANamp Bacteria DNA Kit (Tiangen, China) following the manufacturer's instructions. The Illumina paired-end libraries with an average of 300 bp inserts were prepared from bacterial genomes and sequenced using the Illumina MiSeq sequencer (Illumina, San Diego, USA). The NGS QC Toolkit v2.3.1 (Patel and Jain, 2012) was used to screen the high-quality (HQ) read pairs with the following parameters: the cut-off read length for HQ was 70% and the cut-off quality score was 20. HQ filtered reads were then *de novo* assembled using Velvet (Zerbino and Birney, 2008) with various k-mers, and the best resulting assembly was chosen based on contiguity statistics. Finally, the completeness of the newly assembled genomes presented here was evaluated by the CheckM package (Parks et al., 2015).

### Taxonomic and functional analysis

The 16S ribosomal RNA (rRNA) gene sequences dispersed in new genome assemblies were obtained using RNAmmer (Lagesen et al., 2007) in order to determine the bacterial phylogeny. Similar sequences were identified using the online BLAST search tool. Multiple sequence alignment for the complete 16S rRNA sequences of the novel strains and similar sequences retrieved from GenBank was performed using ClustalX v1.81. The nucleotide pairwise genetic distances were calculated by implementing the Tamura-Nei model of nucleotide substitution. The maximum likelihood tree was constructed from the ClustalX results using MEGA v5.05 (Tamura et al., 2011). The node support was evaluated using 1,000 bootstrap replications. To further infer the phylogenetic relationship, *in silico* DNA-DNA hybridization was performed using JSpecies v1.2.1 software (Richter and Rosselló-Móra, 2009) by calculating the average nucleotide identity (ANI) based on the BLAST algorithm (ANIb, Goris et al., 2007) and tetranucleotide frequency correlation coefficient (TETRA, Teeling et al., 2004). The ANI calculation was conducted using the default and evaluated parameters: sequence identity (%) ≥ 30%, alignment (%) ≥ 70%, and query length = 1,020 bp. In addition, the default values of –*X*(150), −*q* (−1), −*F* (F), −*e* (1e^−15^), and –*a* (2) were applied for the ANIb calculation.

The prediction of putative protein-coding sequences (CDS) and automatic annotation were achieved using the NCBI Prokaryotic Annotation Pipeline. In-house Perl scripts were used to extract protein sequences from the GenBank files; these sequences were then aligned against the specialized databases, such as the extended Clusters of Orthologous Groups (COG, Franceschini et al., 2013), with a BLASTP algorithm and an *E*-value cut-off of 1e^−5^. Visualization was performed using HemI (Deng et al., 2014) for the percentage of CDS assigned to the COG categories. The metabolic potentials of all of the strains were reconstructed using the KEGG Automatic Annotation Server (KAAS) with the BLAST algorithm against the manually curated KEGG GENES database (Moriya et al., 2007). ISFinder (Siguier et al., 2006) was used to identify the putative transposable elements, including transposons and insertion sequences. Additionally, tRNA genes were identified by tRNAscan-SE (Lowe and Eddy, 1997).

### Identification of orthologous proteins and comparison of genome architectures

A BLASTP all-vs-all comparison of protein sequences extracted from the GenBank files was conducted. BLAST results with a tabular format were used to identify the orthologous groups implementing the program Pan-genome Ortholog Clustering Tool (PanOCT) v3.18 (Fouts et al., 2012). The following parameters were applied: *E* ≤ 0.001, percent identity ≥ 30, and length of match ≥ 65 bp, reference to a previous study (Zhang and Sievert, 2014). In consideration of the lineage-specific expansions (Carretero-Paulet et al., 2015), the transposable elements were filtered out in this study. Finally, the comparative results were manually checked.

Protein sequences shared by all strains and unique to individual species were extracted from the PanOCT results and they were then aligned against the extended COG as described above. Furthermore, the intra-species divergence was investigated by BLASTN-based whole genome comparisons with the following parameters: *E* ≤ 1e^−5^ and sequence identity ≥ 50%. Circular visualization of genomic data was performed using Circos software (Krzywinski et al., 2009).

### Availability of supporting data

The Whole Genome Shotgun projects have been deposited at DDBJ/ENA/GenBank under the accession MPOJ00000000 (*Leptospirillum ferriphilum* DX) and MPOK00000000 (*L. ferriphilum* ZJ). The versions described in this paper are version MPOJ01000000 and MPOK01000000. Additionally, the data sets regarding *Acidithiobacillus caldus* DX (LZYE00000000), *A. caldus* ZJ (LZYG00000000), *S. thermosulfidooxidans* DX (MDZD00000000), and *S. thermosulfidooxidans* ZJ (MDZF00000000) are available in the NCBI repository. The corresponding versions described in this article are version LZYE02000000, LZYG02000000, MDZD02000000, and MDZF02000000, respectively.

## Results

### Phylogeny of newly sequenced strains and overall genome statistics

**Figure 1:**
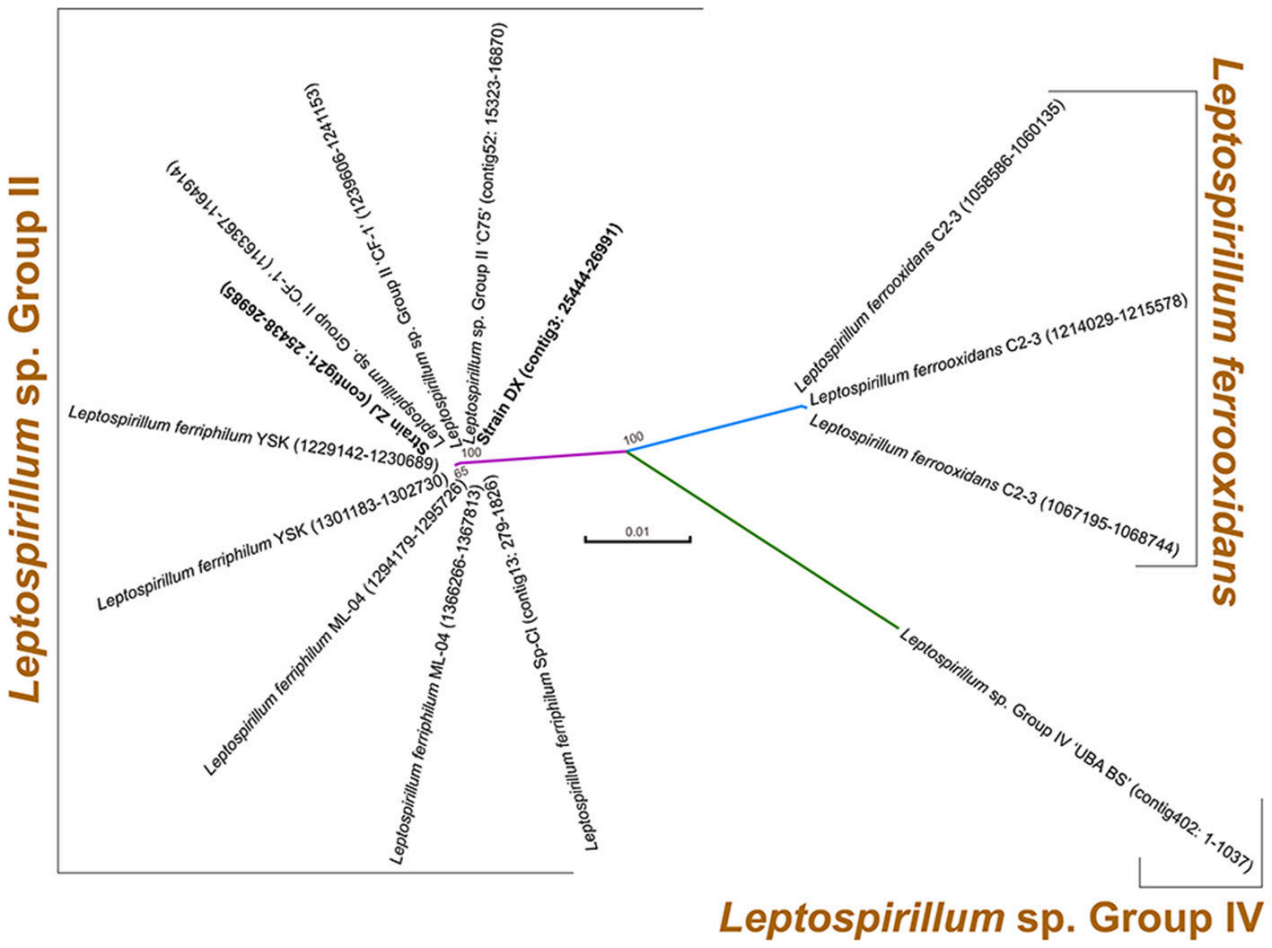
**Phylogenetic tree based on 16S rRNA genes of two newly sequenced strains and other known ***Leptospirillum*** spp**. For each strain, 16S rRNA gene sequence was extracted from complete or draft genome using RNAmmer, and its genomic locus was shown.

Two novel strains in this study were isolated from Dexing Copper Mine and Zijinshan Copper Mine. There were 16S rRNA gene sequences that were extracted from these two newly sequenced genomes using RNAmmer, and they were then used for the identification of phylogeny. Phylogenetic analysis showed that the novel strains were clustered on a distinct branch within *Leptospirillum* sp. Group II, but they were obviously distinguished from other *Leptospirillum* groups, suggesting a close relationship between new isolates and *Leptospirillum* sp. Group II (Figure 1). Accordingly, eight *Leptospirillum* genomes that are available in the GenBank database were chosen for phylogenomic analysis (Table 1). The phylogenetic relationship among all putative or recognized *Leptospirillum* strains was inferred by the comparison of the average nucleotide identity (ANI) based on BLAST (ANIb) and tetranucleotide frequency correlation coefficient (TETRA). The values of ANIb (≥ 97.96%) and TETRA (≥ 0.999) that were evaluated by JSpecies strongly indicated that strains DX and ZJ analyzed in our study were very closely related to *L. ferriphilum* strains but separate from other *Leptospirillum* spp. (Table 1), further supporting the prior notion of 16S rRNA gene-based phylogenetic analysis. In addition, these two newly sequenced strains (*L. ferriphilum* DX and ZJ) shared high values of ANIb (98.56) and TETRA (0.999) with each other (Table 1). Collectively, we inferred that the novel strains DX and ZJ inspected in this study were phylogenetically affiliated with *L. ferriphilum*.

**Table 1:** **Genome-based phylogenetic indicators of two novel strains compared to other recognized ***Leptospirillum*** spp**.

**Organism**	**Accession ID**	ANIb (%)^*^		TETRA^*^	
		**DX**	**ZJ**	**DX**	**ZJ**
Strain DX	MPOJ00000000	—	98.56	—	0.999
Strain ZJ	MPOK00000000	98.56	—	0.999	—
*Leptospirillum ferriphilum* YSK	CP007243	98.54	98.94	0.999	0.999
*Leptospirillum ferriphilum* ML-04	CP002919	98.53	97.97	0.999	0.999
*Leptospirillum ferriphilum* Sp-Cl	LGSH01000000	97.96	98.65	0.999	0.999
*Leptospirillum* sp. Group II ‘CF-1’	CP012147	96.80	96.94	0.996	0.996
*Leptospirillum* sp. Group II ‘C75’	AIJM01000000	96.77	96.91	0.999	0.998
*Leptospirillum* sp. YQP	LIEB00000000	84.42	84.42	0.576	0.578
*Leptospirillum* sp. Group IV ‘UBA BS’	AURA01000000	67.29	66.99	0.870	0.863
*Leptospirillum ferrooxidans* C2-3	NC_017094	66.63	66.35	0.872	0.874

* *Values below the thresholds of 95% (ANIb) and 0.99 (TETRA) suggest that the two of strains belong to different species (Richter and Rosselló-Móra, 2009). Leptospirillum genus was recognized recently to harbor four groups, including Group I (L. ferrooxidans), Group II (L. rubarum and L. ferriphilum), Group III (L. ferrodiazotrophum), and Group IV*.

Genomic features of *L. ferriphilum, A. caldus* (Zhang et al., 2016b), and *S. thermosulfidooxidans* (Zhang et al., 2017), the latter two of which were also used as part of this study, are summarized in Table 2. Each genome had between 20 to 396 contigs with coverage ranging from 52 to 165 ×. Genome completeness assessed by CheckM suggested the near-complete genomes of all six strains, although the genome coverage of *L. ferriphilum* ZJ was relatively low compared to the other strains. *A. caldus* and *S. thermosulfidooxidans* had larger genomes (between 3.12 and 3.18 Mbp) than *L. ferriphilum* (between 2.34 and 2.36 Mbp). *A. caldus* strains with higher GC contents (DX with 61.01% and ZJ with 61.00%) were observed, while the GC contents in *L. ferriphilum* and *S. thermosulfidooxidans* ranged from 48.47 to 54.70%.

**Table 2:** **Comparison of genomic and phenotypic features of co-occurring bacteria isolated from two distinctive copper mines**.

**Organism**	***Leptospirillum ferriphilum* DX**	***Acidithiobacillus caldus* DX**	***Sulfobacillus thermosulfidooxidans* DX**	***Leptospirillum ferriphilum* ZJ**	***Acidithiobacillus caldus* ZJ**	***Sulfobacillus thermosulfidooxidans* ZJ**
Geographic origin	Copper mine tailings, Jiangxi, China			Copper mine tailings, Fujian, China		
Nutritional type	Chemoautotrophic	Chemoautotrophic	Mixotrophic	Chemoautotrophic	Chemoautotrophic	Mixotrophic
Genome status	Draft	Draft	Draft	Draft	Draft	Draft
Accession number	MPOJ00000000	LZYE00000000	MDZD00000000	MPOK00000000	LZYG00000000	MDZF00000000
Coverage	165×	95×	132×	52×	76×	136×
Completeness (%)	93.15	98.76	99.00	93.02	98.14	99.00
Genome size (Mb)	2.36	3.12	3.18	2.34	3.14	3.18
Number of contigs	30	390	33	104	386	20
GC content (%)	54.51	61.01	48.47	54.70	61.00	48.47
N50 length (bp)	118,116	22,157	171,016	54,724	18,308	452,891
N90 length (bp)	51,004	4, 321	52,886	12,896	2,291	108,775
**RNA GENES**						
5S rRNA count	1	1	1	1	1	2
16S rRNA count	1	1	1	1	1	1
23S rRNA count	1	1	1	1	1	1
tRNA count	46	46	51	46	46	51
Putative CDS	2,342	2,841	2,958	2,355	2,864	2,958
COG	1,777 (75.88%)	2,250 (79.20%)	2,454 (82.96 %)	1,790 (76.01%)	2,274 (79.40%)	2,460 (83.16%)
Reference	This study	Zhang et al., 2016b	Zhang et al., 2017	This study	Zhang et al., 2016b	Zhang et al., 2017

All of the genome assemblies had the full suite of tRNA that covered all of the 20 amino acids. Bacterial genomes were predicted to contain between 2,342 and 2,958 protein-coding sequences (CDS). The Clusters of Orthologous Groups (COG) annotation suggested that between 75.88 and 83.16% of the CDS were matched to putative proteins with known functions (Table 2). Of the 25 COG categories, CDS were assigned to 21 (*L. ferriphilum* and *S. thermosulfidooxidans*) or 22 (*A. caldus*) of them. In addition to COG categories [S] (Function unknown) and [R] (General function prediction only), “Energy production and conversion (C),” “Amino acid transport and metabolism (E),” and “Cell wall/membrane/envelope biogenesis (M)” were commonly abundant in all of the genomes (Figure S1). It is important to note that the proportion of CDS assigned to “carbohydrate transport and metabolism (G)” in *S. thermosulfidooxidans* (5.17% in DX and 5.21% in ZJ) was relatively large compared to the other two species (between 2.83 and 3.12%; Figure S1).

### Identification of core genes and flexible genes

**Figure 2:**
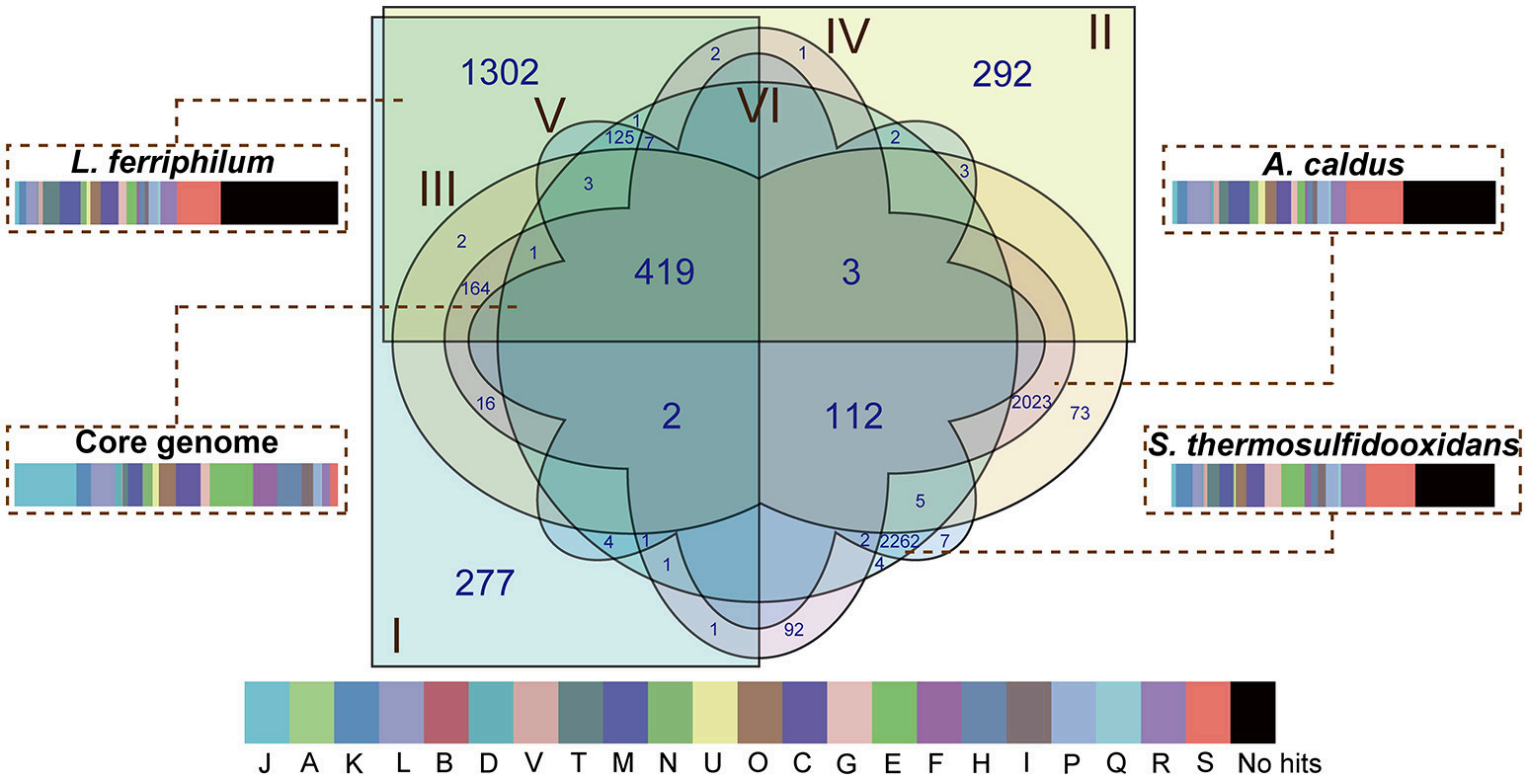
**Venn diagram depicting orthologous and non-orthologous genes**. *L. ferriphilum* DX (I), *L. ferriphilum* ZJ (II), *A. caldus* DX (III), *A. caldus* ZJ (IV), *S. thermosulfidooxidans* DX (V), and *S. thermosulfidooxidans* ZJ (VI) are shown in different colors. The numbers shown in the Venn diagram indicate the number of orthologous or strain-specific genes. If no number is given in certain patterns, it suggests there is no orthologous protein among these strains. The core genome and unique genes in each species were used for functional analysis based on COG classification. The descriptions of the COG categories are provided in Figure S1. The width of rectangle indicates the percentage of CDS compared to the entire genome of individual strains.

There were 8,114 CDS that were clustered using the PanOCT with a 30% sequence identity cut-off in order to infer the shared and flexible genes among the tested strains. Comparative analysis showed that 419 putative orthologous genes were shared by all of the strains (Figure 2), representing the low proportions compared to the entire CDS of the individual genome: 17.89% in *L. ferriphilum* DX, 17.79% in *L. ferriphilum* ZJ, 14.75% in *A. caldus* DX, 14.63% in *A. caldus* ZJ, 14.16% in *S. thermosulfidooxidans* DX, and 14.16% in *S. thermosulfidooxidans* ZJ. These shared genes were mainly associated with “translation, ribosomal structure, and biogenesis (J),” “amino acid transport and metabolism (E),” “energy production and conversion (C),” and “nucleotide transport and metabolism (F).” Aside from the core genes that are common in all of the strains, the flexible genes, including genes present in some but not all of the genomes and strain-specific genes unique to the individual genomes are also depicted (Figure 2). Notably, the CDS only shared by each species (1,302 in *L. ferriphilum*, 2,023 in *A. caldus*, and 2,262 in *S. thermosulfidooxidans*) were much more than the core genes, clearly suggesting the genomic difference among these organisms. Of these sequences, the COG classification revealed that many were annotated as hypothetical proteins or proteins with unknown functions. In addition, the CDS involved in the COG categories [M] (cell wall/membrane/envelope biogenesis) and [C] were numerically significant, highlighting the distinguishing features among these species. Similar to the results shown in Figure S1, the percentage of the CDS related to the COG category [G] in the *S. thermosulfidooxidans* species (5.13%) were larger than that in the others (2.53% in *L. ferriphilum* and 1.93% in *A. caldus*). Of genes unique in *S. thermosulfidooxidans*, many were involved in the biosynthesis of amino acid, such as cysteine, arginine, glycine, as well as alanine (unpublished).

**Figure 3:**
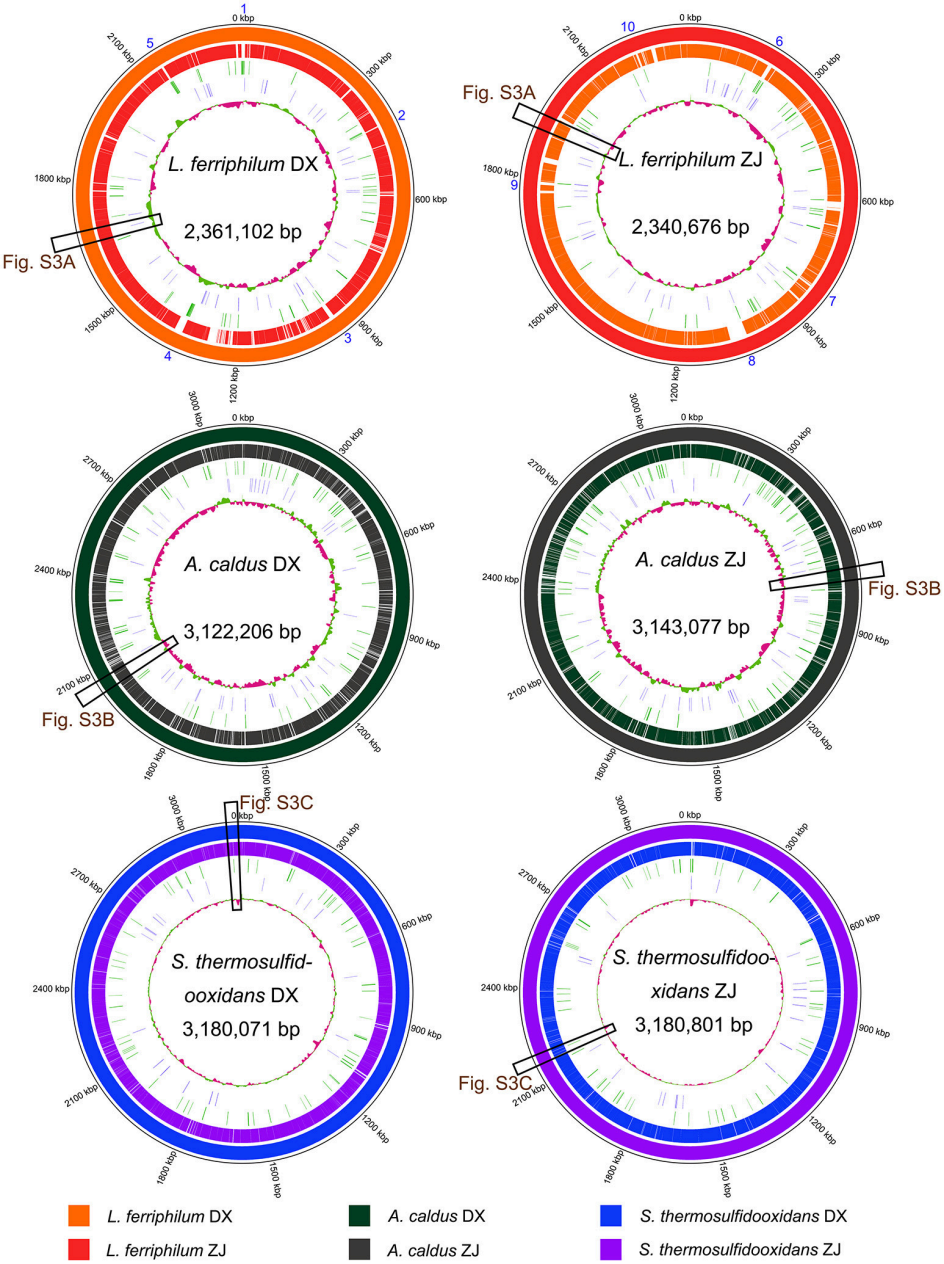
**Reference-based whole genome comparisons and visualization of each strain**. GC contents of individual genomes are indicated in the center of the figure. In addition, tRNA and transposases are shown on the second and the third rings from the inside. Matches to the reference genomes (≥ 50% sequence identity) are shown with different colors. Cross references showing further analysis of certain genomic regions are linked to other figures. Detailed description for genomic regions 1–10 in *L. ferriphilum* isolates are shown in Table S1.

BLASTN-based whole genome comparisons of these targeted strains isolated from distinct areas were performed and visualized using Circos (Figure 3) to acquire some insights into intra-species differences. An examination of the architecture and global structural genomes revealed that a number of non-shared genomic regions were found between the strains belonging to the same species, although they shared a high-sequence identity. Further inspection revealed that many genes distributed in the genomic regions (sections 1–10 in Figure 3) of each *L. ferriphilum* strain were predicted to encode putative proteins with unclear functions (Table S1). In addition, several mobile genetic elements, such as transposase, integrase, and phage-associated protein, were identified in the genomic neighborhoods. Similar results were also reported in *A. caldus* (Zhang et al., 2016b) and *S. thermosulfidooxidans* (Zhang et al., 2017).

### Comparison of inferred metabolic profiles

The KAAS online server was employed to investigate the metabolic potential of the microorganisms in this study (Table S2). In general, four types of metabolism having the most abundant genes in all of the strains were: “carbohydrate metabolism,” “amino acid metabolism,” “energy metabolism,” and “metabolism of cofactors and vitamins.” Metabolism-related CDS in *S. thermosulfidooxidans* with a larger genome (1,124 in DX and 1,125 in ZJ) were more than that in the other two species (between 818 and 885). Furthermore, the key metabolisms of each genome are listed and comparisons are presented in Table 3. In the following section, we discuss the metabolic differences among the species.

**Table 3:** **Overall comparisons of the sequenced genomes of ***L. ferriphilum*** DX (1), ***L. ferriphilum*** ZJ (2), ***A. caldus*** DX (3), ***A. caldus*** ZJ (4), ***S. thermosulfidooxidans*** DX (5), and ***S. thermosulfidooxidans*** ZJ (6) with reference to key metabolic potentials**.

**Metabolic pathways**	**1**	**2**	**3**	**4**	**5**	**6**
**CARBON ASSIMILATION**						
Calvin cycle						
RuBisCO			2	2	1	1
RubisCO-like protein	1	1	1	1		
Carboxysome						
TCA cycle	i	i	i	i		
rTCA cycle						
Glycolysis / Gluconeogenesis						
Glycolic acid assimilation						
**NITROGEN UPTAKE**						
Ammonium uptake						
Dissimilatory nitrate reduction						
Assimilatory nitrate reduction						
Urease complex						
**SULFUR METABOLISM**						
Sulfur oxygenase reductase			1	1	2	2
Sox system			2	2		
**IRON OXIDATION**						
Cyc_572_						
Cyc_579_	2	2				
*cbb_3_*-type cytochrome oxidase						
*bc_1_* complex	2	2				
Sulfocyanin					2	2
*aa_3_*-type cytochrome oxidase						

*White cells and blue cells represent absence and presence of the metabolic enzymes or metabolic pathways in individual genomes, respectively. Additionally, the number of copies of gene/operon was highlighted in white letterings; i, incomplete pathway*.

#### Central carbon metabolism

As previously noted by Berg et al. (2010), six autotrophic carbon fixation mechanisms that assimilate CO_2_ into cellular material have been well-documented, including the Calvin-Benson-Bassham (CBB) cycle, the reductive tricarboxylic acid (rTCA) cycle, the reductive acetyl-coenzyme A pathway, the 3-Hydroxypropionate bicycle, the hydroxypropionate-hydroxybutyrate cycle, and the dicarboxylate-hydroxybutyrate cycle. Ribulose-1,5-bisphosphate carboxylase/oxygenase (RuBisCO), one of the key enzymes associated with the first rate-limiting step for the CBB cycle, has been classified into four forms: I-IV (Zhang et al., 2016c). In previous studies, the CBB cycle was considered to be the way to fix carbon in *Leptospirillum* genus (Coram and Rawlings, 2002). Our present results showed that no gene encoding putative RuBisCO was identified in *L. ferriphilum* isolates, but non-canonical RuBisCO-like protein was predicted (Table S3). However, *L. ferriphilum* genomes were predicted to harbor a full suite of genes encoding canonical enzymes, which were involved in the rTCA cycle for carbon fixation. Similar results could be also found in several other reviews and papers (Levicán et al., 2008; Mi et al., 2011; Zhang et al., 2016c). In particular, genes encoding pyruvate ferredoxin oxidoreductase and 2-oxoglutarate ferredoxin oxidoreductase were predicted to exist in a common genome region (Figure S2A). In contrast, *A. caldus* strains were reported to harbor distinct forms of RuBisCO, and *S. thermosulfidooxidans* only had type I RuBisCO. For the *A. caldus* genomes, genes encoding type I RuBisCO were located in a gene cluster involved in carboxysome formation (Figure S2B). Notably, *A. caldus* isolates were predicted to harbor a carboxysome-associated carbonic anhydrase; however, no genome region related to carboxysome was identified in *S. thermosulfidooxidans*, despite the existence of a gene encoding the putative carbonic anhydrase.

All of the isolates were also anticipated to share a variety of genes predicted to be involved in the synthesis of lipopolysaccharides, peptidoglycan, and, potentially, exopolysaccharides (Table S3). In all of the isolates, the accumulated 3-phosphoglycerate from carbon fixation was predicted to be converted via glycolysis/gluconeogenesis to produce the major precursors (glucose-6-phosphate and glucose-1-phosphate) for the biosynthesis of cell envelope polysaccharides. In light of an earlier report using “*Ferrovum*” genomes (Ullrich et al., 2016a), a core set of genes that are potentially associated with amino sugar and nucleotide sugar metabolism (ko00520) were predicted in all of the investigated strains (Table S3). A variety of intermediate metabolites, such as UDP-glucose, UDP-glucuronate, UDP-galactose, and UDP-N-acetyl-glucosamine, within these metabolic processes might be potential precursors for the biosynthesis of cell envelope polysaccharides in all of the strains. Several genes that are potentially involved in the formation of precursors, such as UDP-mannose and UDP-N-acetyl-mannosamine, were only identified in the *S. thermosulfidooxidans* genomes, while *L. ferriphilum* and *A. caldus* were found to share the potential to produce alternative precursors, such as ADP-glucose and UDP-galacturonate. Furthermore, only the *A. caldus* strains appeared to catalyze GDP-mannose to GDP-4-dehydro-6-deoxy-D-mannose by GDP-mannose 4,6-dehydratase (EC 4.2.1.47).

*Leptospirillum ferriphilum* and *Acidithiobacillus caldus* were found to harbor a slightly larger enzyme repertoire related to the aforementioned biosynthesis, suggesting the production of additional macromolecules with species-specific monosaccharide compositions in comparison with *S. thermosulfidooxidans*. For mechanisms potentially involved in the export of cell envelope polysaccharides, the ABC-2-type polysaccharide transport system was identified in all strains, while the Lpt-type lipopolysaccharide export system was found solely in *L. ferriphilum* and *A. caldus*. Compared to the other microorganisms (Haft et al., 2006; Craig et al., 2011; Ullrich et al., 2016a), only *L. ferriphilum* strains shared the genes predicted to encode the PEP-CTERM exosortase system that was presumed to be involved in the export of exopolysaccharides during biofilm formation. In several earlier studies, biofilm was proposed to provide a reaction space between the sulfide surfaces and bacterial cells, thereby accelerating sulfide mineral dissolution (Watling, 2006; Rohwerder and Sand, 2007; González et al., 2013).

*Acidithiobacillus caldus* and *Leptospirillum ferriphilum* were predicted to exhibit an incomplete TCA cycle (Table S3). However, *S. thermosulfidooxidans* strains harbored a full suite of genes encoding the tricarboxylic acid cycle (TCA), which was thought to be essential for heterotrophic growth (Wood et al., 2004). In addition, *S. thermosulfidooxidans* strains exhibited the potential ability to degrade glycolic acid, as genes encoding putative glycolate oxidase and malate synthase were found in both genomes (Table S3). In particular, *S. thermosulfidooxidans* strains were predicted to harbor genomic regions potentially associated with glycolate oxidase (Figure S2C). A pairwise comparison of these gene clusters revealed the identical order and orientation of all of the genes, as well as the nucleotide sequence identities of up to 100%, suggesting that the gene clusters involved in glycolate oxidase might evolve from a common ancestor.

As for comparison and analysis, genome sequences of these six strains were used for the identification of carbohydrate-active enzymes (CAZymes), which represented an enormous number of enzymes that were responsible for the assembly, modification, and breakdown of oligo- and polysaccharides (Lombard et al., 2013). As shown in Table S4, the gene repertoire of potentially encoding CAZymes, including glycoside hydrolases (GHs), glycosyltransferases (GTs), polysaccharide lyases (PLs), carbohydrate esterases (CEs), auxiliary activities (AAs), and carbohydrate-binding modules (CBMs), were identified in all of the genomes. Apparently, more abundant CAZymes were identified in the *S. thermosulfidooxidans* genomes (156 in DX and 157 in ZJ) compared to the others (between 80 and 92), especially the GHs that are responsible for the hydrolysis of glycosidic bonds and the CEs that hydrolyze the carbohydrate esters. The most abundant GH classes in the *S. thermosulfidooxidans* isolates were strongly biased toward the catabolism of oligo- or polysaccharides, such as chitinase (GH18), cellobiohydrolase (GH74), glucoamylase (GH15), and α-amylase (GH13).

Similar to an earlier study (Yelton et al., 2013), another potential organic carbon source for the *S. thermosulfidooxidans* strains might be the lipids from the lysed cells. They were predicted to harbor a full set of putative proteins involved in fatty acid degradation (Table S3), including long-chain acyl-CoA synthetase, acyl-CoA dehydrogenase, enoyl-CoA hydratase, and acetyl-CoA acyltransferase. Additionally, lactate permease and lactate utilization of protein B/C was identified in the *S. thermosulfidooxidans* isolates (Table S3), suggesting their heterotrophic growth on lactate.

#### Nitrogen uptake

*Acidithiobacillus caldus* strains harbor the complete genes required for dissimilatory nitrate reduction, which were observed as *NarGHJI* and *nirBD* operons (Table S3). Thus, it was hypothesized that the *A. caldus* strains in this study might utilize nitrate and nitrite as electron acceptors under anaerobic environmental conditions. As for assimilatory nitrate reduction, *A. caldus* was predicted to harbor *narA* potentially as an encoding assimilatory nitrate reductase catalytic subunit; however, there was no NasB subunit, which was reported to transfer electrons from NADH to nitrate (Lin and Stewart, 1997), thereby making the electron donor unclear. Additionally, no *nirA* gene was identified in the *A. caldus* genomes. In contrast, *S. thermosulfidooxidans* share the set of genes that encode assimilatory nitrate reduction. We identified the genes encoding the assimilatory nitrate reductase catalytic subunit (NasA) and ferredoxin-nitrite reductase (NirA). Although putative genes potentially encoding dissimilatory nitrite reductase small subunit (NirD) and ferredoxin-nitrite reductase (NirA) were found in the *L. ferriphilum* genomes, their functional roles need to be studied further. Additionally, all of the strains share the potential to utilize ammonium via the Amt family transporter (Table S3), and to assimilate it into the central metabolic pathways via glutamine synthetase or glutamate synthase. Given that dissimilatory nitrate reduction and assimilatory nitrate reduction were absent in *L. ferriphilum*, they might utilize ammonium as the sole nitrogen source. In addition, *S. thermosulfidooxidans* was the only species that contains gene cluster encoding urease subunits (UreABC) and accessory proteins (UreDEFG; Table S3). Accordingly, it seems that *S. thermosulfidooxidans* could utilize extracellular urea, although there is still no direct evidence showing the existence of these compounds in the bioleaching heaps.

#### Iron-sulfur cycling

**Figure 4:**
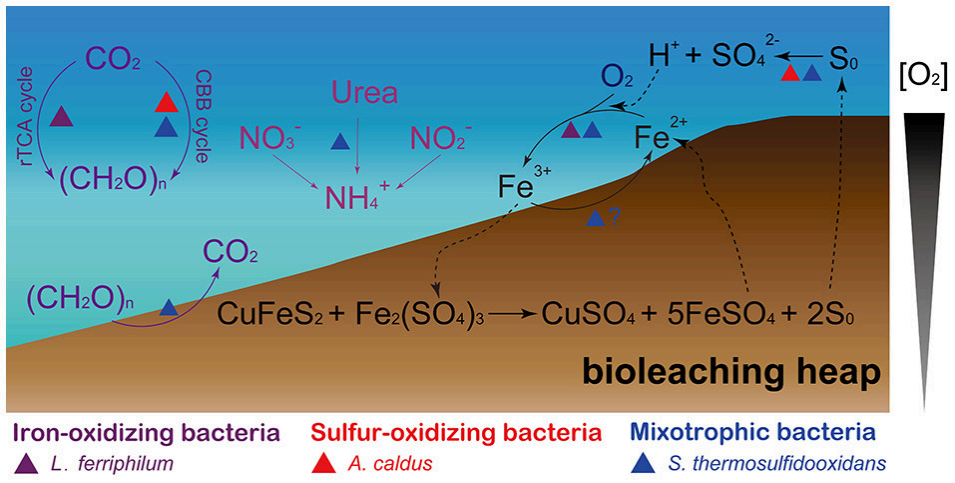
**Schematic representation showing the microbe-mediated biogeochemical cycle of main elements (carbon, nitrogen, iron, and sulfur) in bioleaching heaps**. The figure was adapted from Johnson (1998), Baker and Banfield (2003), and Chen et al. (2016).

Both *L. ferriphilum* and *S. thermosulfidooxidans* are demonstrated iron oxidizers (Guo et al., 2014; Justice et al., 2014; Zhang et al., 2016c). These two species were predicted to harbor membrane-associated *c*-type cytochromes that are potentially involved in iron oxidation. In *L. ferriphilum*, the electrons might be transferred from an outer-membrane cytochrome *c* (Cyc_572_) to a periplasmic cytochrome *c* (Cyt_579_), and then across periplasmic cytochromes *c* either reducing oxygen via *cbb*_3_-type terminal oxidase (downhill), or reducing NAD^+^ passing through the *bc*_1_ complex, quinone pool, and NADH dehydrogenase (uphill, Bonnefoy and Holmes, 2012). Similar to Cyt_579_ in *L. ferriphilum*, the periplasmic sulfocyanins identified in *S. thermosulfidooxidans* (Table S3) were postulated to be the branch point at which electrons derived from membrane *c*-type cytochromes might be channeled either downhill or uphill. As for the uphill pathway, the *bc*_1_ complex in *Leptospirillum* spp. was proposed to utilize the proton motive force (PMF) to push electrons uphill against the thermodynamically unfavorable gradient (Bonnefoy and Holmes, 2012). Herein, the outflux of protons catalyzed by the *cbb*_3_-type terminal oxidase contributes to the generation of the PMF during electron transfer to oxygen via a downhill pathway. However, genes encoding the putative *bc*_1_ complex were absent in *S. thermosulfidooxidans*. It seems that this species may not require reverse electron transfer to generate reduction equivalents (e.g., NADH and NADPH), but depend on the oxidation of organic compounds, such as TCA cycle, oxidative pentose phosphate pathway, to produce these compounds, since it was reported to be mixotroph capable of the assimilation of both inorganic and organic carbon compounds. In addition, *A. caldus* and *S. thermosulfidooxidans* analyzed here were replete with metabolic enzymes related to sulfur oxidation (Table S3), and they have the ability to oxidize minerals, which produce sulfuric acid (Figure 4). Their activities facilitate the dissolution of base metals in certain acid-soluble sulfide minerals, such as chalcocite, via an oxidative route (Johnson and Hallberg, 2003). Oddly, gene encoding putative thiosulfate:quinone oxidoreductase (TQO) was absent in both *A. caldus* strains, while the corresponding gene was identified in the homologous strains such as SM-1 and ATCC 51756 (Zhang et al., 2016b). In addition, gene encoding sulfate adenylyltransferase (SAT) was not found in *S. thermosulfidooxidans* genomes analyzed in this study, however, the homologous strain ST was reported to harbor the *sat* gene (Guo et al., 2014; Zhang et al., 2017). In *A. caldus* (Chen et al., 2012), electrons from TQO, sulfide quinone reductase, sulfur oxidizing protein, and heterodisulfide reductase were mediated by the quinol pool, and then (i) either were transferred to oxygen via *bd*-type or *bo*_3_-type terminal oxidases, (ii) or were transferred to NADH complex I (Table S3). Similarly, genes encoding putative *bd*-type terminal oxidase and NADH complex I were identified in *S. thermosulfidooxidans* genomes.

## Discussion

### General comparisons of the gene repertoire among bacterial genomes

The overview of bacterial genomes is shown in Table 2. Compared to the others, the GC contents of the *A. caldus* genomes were much higher; a plausible explanation for this finding is that the optimal growth temperature (*T*_opt_) is regarded as one of the environmental factors that positively influences genomic GC content in prokaryotes (Musto et al., 2004, 2006) given that *A. caldus* is the primary sulfur oxidizer in bioleaching operations at temperatures above 40°C (Acuña et al., 2013). Nevertheless, this inference was widely divergent from our recollection since the moderate thermophile *S. thermosulfidooxidans* harbors relatively low GC contents. In fact, the GC contents of the bacterial genomes varied dramatically, and they were influenced by multiple factors (Hildebrand et al., 2010), such as genome size (Bentley and Parkhill, 2004), environment (Foerstner et al., 2005), nitrogen utilization (Mcewan et al., 1998), and aerobiosis (Naya et al., 2002). However, more evidence should be provided to further determine whether the relatedness between the genomic GC contents and multifactorial mechanisms is a stochastic process or a result of natural selection.

An analysis of the gene contents showed that all of the genomes were predicted to harbor a large number of CDS that were potentially assigned to the COG categories [C], [E], and [M]. This finding was similar to our previous study (Zhang et al., 2016a). The COG categories [C] and [E] are involved in the metabolic pathways of energy and amino acids, and COG category [M] is related to the synthesis of cell membranes, membrane channel proteins, and extracellular polymeric substances. Thus, we infer that the bacterial lifestyle requires efficient utilization of energy and nutrients from external environments and specialized cellular structures to adapt to changing environments. In addition, the abundant CDS in these bacterial genomes were assigned to COG category [L] (replication, recombination, and repair), which contains many enzymes associated with DNA repair. The habitats that these acidophilic microorganisms inhabit are characterized by extreme acidity and an abundance of heavy metals. When cell damage occurs, the corresponding proteins may play an important role in DNA repair (Mi et al., 2011; Chen et al., 2013). Of note, many CDS assigned to COG category [E] were only identified in *S. thermosulfidooxidans* genomes. We therefore hypothesize that *S. thermosulfidooxidans* strains exhibited additional abilities to synthesize amino acids, suggesting that they depend more heavily on organic compound assimilation than the others.

### Inter- and intra-species genome comparison

A comparative survey of three species (*S. thermosulfidooxidans, L. ferriphilum*, and *A. caldus*) that are commonly recognized in many acidic and sulfur-containing environments was performed. Each species was distinctively different from the others, which strongly suggests the genomic differences among these isolates. It is important to note that the number of CDS associated with carbohydrate transport and metabolism in *S. thermosulfidooxidans* was relatively large in comparison with its counterparts. Hence, we interpreted this as an indication that the *S. thermosulfidooxidans* strains were mixotrophic acidophiles that are capable of assimilating of inorganic and organic carbon. Collectively, the findings presented herein imply that genomic and functional differentiation of these acidophilic species in the same community might allow them to co-exist.

Acidophilic bacteria analyzed in this study were isolated from different tailings samples located at Dexing Copper Mine and Zijinshan Copper Mine. Since mobile genetic elements, such as transposases and integrases, were recognized as the signatures of potential horizontal gene transfer (HGT) events (Waack et al., 2006; Juhas et al., 2009; Acuña et al., 2013), the identification of mobile genetic elements in genomic neighborhoods of *L. ferriphilum* strains implied that the bacterial genomes were likely to undergo the HGT event, resulting in the coincidental acquisition of novel functionalities that might be advantageous under certain conditions (Gogarten et al., 2002). The finding was similar to our earlier studies concerning the intra-species divergence of *A. caldus* (Zhang et al., 2016b) and *S. thermosulfidooxidans* (Zhang et al., 2017) at the genome level. Thus, in this study we underscore the important role of HGT in shaping the genetic content of bacterial genomes and contributing to intra-species differentiation.

### Differences in metabolic profiles of acidophilic microorganisms

Our genomic analysis focused on the differences of key metabolic pathways, including carbon, nitrogen, iron, and sulfur metabolism (Figure 4). The species with different metabolic capacities may efficiently utilize nutrients or other resources in the same community, likely allowing them to co-exist in bioleaching heaps and thus, avoid competitive exclusion.

#### Central carbon metabolism

An earlier study revealed that *Acidithiobacillus* spp. and/or *Leptospirillum* spp. were observed to be the major carbon fixers in acid mine drainage (AMD) communities (Chen et al., 2015). Previous studies showed detailed evidence supporting the fixation of carbon dioxide via the classical CBB cycle in *A. caldus* (You et al., 2011; Zhang et al., 2016b) and *S. thermosulfidooxidans* (Guo et al., 2014; Justice et al., 2014; Zhang et al., 2017). Apart from autotrophic growth, *S. thermosulfidooxidans* isolates have been reported to grow heterotrophically via the catabolism of various organic carbon compounds (Guo et al., 2014; Justice et al., 2014). Unlike *S. thermosulfidooxidans, A. caldus* was predicted to elevate the concentration of carbon dioxide near RuBisCO by a carboxysome-associated carbonic anhydrase, which was responsible for the conversion of accumulated cytosolic bicarbonate into CO_2_ (Zhang et al., 2016e). Thus, carboxysome within *A. caldus* strains might be a critical component that contributes to their ability to grow autotrophically through the efficient utilization of scarce carbon availability from acidic environments.

Certain organic compounds, such as cell exudates or lysates that originated from the autotrophic primary producers might be utilized by heterotrophic prokaryotes (Johnson and Hallberg, 2003). Glycolic acid (C00160) was reported to be an exudate originating from autotrophic acidophiles, such as *Leptospirillum* (Justice et al., 2014). In contrast to *Sulfobacillus acidophilus* (Justice et al., 2014), no genes encoding putative isocitrate lyase (EC 4.1.3.1) were identified in *S. thermosulfidooxidans*. Accordingly, we surmise that *S. thermosulfidooxidans* might not have the ability to assimilate isocitrate to malate via glyoxylate bypass, a pathway closely related to the TCA cycle (Yelton et al., 2013). In addition, a gene cluster involved in glycolate oxidase was predicted in *S. thermosulfidooxidans*. A putative transposase identified in its downstream region suggested that this cluster might be introduced by HGT, thereby recruiting a novel ability to degrade the organic carbon (Figure S2C). Taken together, we presented a potential route of glycolic acid assimilation that enables *S. thermosulfidooxidans* to utilize organic acid as an alternative carbon source (Figure S3). In this pathway, glycolate oxidase coupled with malate synthase may degrade glycolic acid to produce malate, and the latter then enters the conventional TCA cycle. Chemolithotrophic acidophiles were sensitive to organic acids in their habitats (Johnson, 1998); therefore, it might be favorable for chemoautotrophs to form a mutualistic relationship with other heterotrophic and/or mixotrophic acidophiles because the latter could effectively detoxify the environments by degrading organic compounds (Johnson and Hallberg, 2003).

In addition, CDS assigned to chitinases (GH18) were found in *S. thermosulfidooxidans*. The GH18 family contained chitinolytic enzymes involved in chitin degradation, and chitin is known to be a substantial component of fungal cell walls. An intriguing explanation for the abundant chitinolytic enzymes in *Trichoderma* is that chitinases are regarded as an integral part of the mycoparasitic lifestyle via lysis of the prey's cell walls (Kubicek et al., 2011). In our study, we could not exclude the possibility that putative chitinases only present in *S. thermosulfidooxidans* might endow them with an additional ability to degrade the lysates of fungi in bioleaching systems. This scenario might be reasonable as fungi were found in several acidic environments, such as AMD ecosystems (Mosier et al., 2016), although their functional roles in bioleaching heaps remain elusive (Cárdenas et al., 2016).

#### Nitrogen uptake

Like other microorganisms in AMD that are characterized by scarce nitrogen availability (Parro et al., 2007; Chen et al., 2015), the habitants in the bioleaching systems may share nitrogen assimilation strategies to cope with the nitrogen-limited conditions. In general, microorganisms commonly utilize atmospheric nitrogen (N_2_), ammonium, nitrite, and nitrate as their inorganic nitrogen sources (Arrigo, 2005). As a member of the *Acidithiobacillus* genus, *A. ferrooxidans* has been predicted to harbor nitrogenase, which is responsible for N_2_ fixation (Levicán et al., 2008; Zhang et al., 2016e). Likewise, nitrogen fixation was also reported in the *Leptospirillum* groups, except for Group II (Goltsman et al., 2013). Under the low nitrogen level, the diazotrophic lifestyle of these bacteria in the microbial communities might be a striking feature to maintain the concentration of nitrogen availability in the habitat environments.

Although the three species analyzed in this study do not have the ability to fix atmospheric nitrogen, they were predicted to utilize environmental ammonium, nitrite, and even nitrate as an alternative nitrogen source. *L. ferriphilum* was predicted to merely harbor the ability of ammonium uptake, but the other two species could utilize other forms of nitrogen, such as nitrate or nitrite as the alternative nitrogen source, thereby, to some extent, avoiding competitive exclusion. Notably, microbial ureases have been reported to hydrolyze environmental urea to produce ammonia and bicarbonate (Mobley and Hausinger, 1989; Ullrich et al., 2016a,b), and ammonia at circumneutral cytoplasmic pH is present as ammonium (Ullrich et al., 2016b). Thus, the *S. thermosulfidooxidans* strains appear to be able to incorporate cytoplasmic ammonium derived from the hydrolysis of urea as a nitrogen source (Figure 4), and utilize the released bicarbonate as an alternative carbon source via carbonic anhydrase.

#### Iron-sulfur cycling

The models for potential iron-sulfur cycling were well-studied in acidic and high-sulfur environments (Johnson, 1998; Baker and Banfield, 2003; Méndez-García et al., 2015; Chen et al., 2016; Zhang et al., 2016e). In these settings, ferrous iron oxidation implemented by iron-oxidizing bacteria, such as *L. ferriphilum* and *S. thermosulfidooxidans*, is the primary biochemical transformation of iron. In addition, the oxidation of sulfide minerals, elemental sulfur, and tetrathionate has been well-documented in *A. caldus* and *S. thermosulfidooxidans* (Mangold et al., 2011; Chen et al., 2012; Guo et al., 2014; Zhang et al., 2016b). Fe(III) under anoxic conditions could accept electrons originating from inorganic (e.g., sulfur and/or hydrogen) and/or organic donors (e.g., glucose and/or glycerol), which occurs in the case of chemolithotrophic and heterotrophic acidophiles, respectively (Méndez-García et al., 2015). When exposed to moisture and air, sulfur-containing mineral ores oxidize spontaneously in situations where molecular oxygen or Fe(III) act as an oxidant (Johnson and Hallberg, 2003). The formation of acidic environments in turn provides advantages to stabilizing Fe(II), even in the presence of atmospheric oxygen (Bonnefoy and Holmes, 2012); this is an opportunity for iron-oxidizing microorganisms inhabiting these settings to utilize Fe(II) as a source of energy.

*Acidithiobacillus caldus* and *Sulfobacillus thermosulfidooxidans* strains used in this study were predicted to lack TQO and SAT encoding genes, respectively. One plausible explanation for this issue was that missing genes might be artifacts of assembly or gene calling procedure. On the other hand, we could not exclude the possibility that gene *tqo* in homologous strains of *A. caldus* (SM-1 and ATCC 51756) and *sat* in homologous strains of *S. thermosulfidooxidans* (ST) might be introduced by HGT. With respect to ferric iron [Fe(III)] reduction, previous studies have shown that *Sulfobacillus* spp. harbored the ability to reduce ferric iron (Melamud et al., 2003; Bogdanova et al., 2006; Johnson et al., 2008). Although Fe(III) reduction activities under oxygen-limiting conditions were observed in acidophilic microorganisms, the determination of a precise enzymatic system is still an important goal for future studies.

Comparative genomics of three typical co-occurring acidophiles in bioleaching heaps yields valuable insights into the differences in gene content, metabolic profiles, and potential functional roles. In short, it could present a scenario whereby certain essential functions are partitioned in co-occurring members of microbial communities, and individual members harboring different metabolic functions generate not merely commensalistic but mutualistic interactions. Thus, it might provide a selective advantage for the efficient utilization of limited resources in their habitats in which they benefit from each other to maintain their respective lifestyle.

## Author contributions

XZ, XL, YL, and HY conceived and designed the experiments. XZ, YX, LM, XG, BM, and HL performed the experiments. XZ analyzed the data. XZ wrote the manuscript. XL, LY, DP, WH, and HY revised the manuscript. All authors read and approved the final manuscript.

### Conflict of interest statement

The authors declare that the research was conducted in the absence of any commercial or financial relationships that could be construed as a potential conflict of interest.

## Supplementary material

Figure S1 **Heat map depicting the percentages of genes assigned to the COG classification in ***A. caldus*** DX (I), ***A. caldus*** ZJ (II), ***L. ferriphilum*** DX (III), ***L. ferriphilum*** ZJ (IV), ***S. thermosulfidooxidans*** DX (V), and ***S. thermosulfidooxidans*** ZJ (VI)**. The five abundant functional categories within all of the bacterial genomes are highlighted in blue.

Figure S2 **Homologous genome regions in individual strains associated with pyruvate ferredoxin oxidoreductase and 2-oxoglutarate ferredoxin oxidoreductase (A)**, carboxysome **(B)**, and glycolate oxidase **(C)**. *porG*, pyruvate ferredoxin oxidoreductase gamma subunit; *porB*, pyruvate ferredoxin oxidoreductase beta subunit; *porA*, pyruvate ferredoxin oxidoreductase alpha subunit; *hp*, hypothetical protein; *forG*, 2-oxoglutarate ferredoxin oxidoreductase gamma subunit; *forB*, 2-oxoglutarate ferredoxin oxidoreductase beta subunit; *forA*, 2-oxoglutarate ferredoxin oxidoreductase alpha subunit; *cbbL*, Rubisco large chain; *cbbS*, Rubisco small chain; *CsoS2*, carboxysome shell protein CsoS2; *CsoSCA*, carboxysome-associated carbonic anhydrase; *CsoS4*, carboxysome shell protein CsoS4; *Tnp*, transposase; *pucR*, PucR family transcriptional regulator; *IclR*, IclR family transcriptional regulator; *glcF*, glycolate oxidase iron-sulfur subunit; *gcl*, glyoxylate carboligase; *hyi*, hydroxypyruvate isomerase; *glxR*, 2-hydroxy-3-oxopropionate reductase; *glcE*, glycolate oxidase FAD binding subunit; *glcD*, glycolate oxidase subunit GlcD.

Figure S3 **Putative route for glycolic acid assimilation within ***S. thermosulfidooxidans*** strains**.

Table S1 **Details for genomic regions of interest in ***Leptospirillum ferriphilum*** strains**.

Table S2 **Comparisons of the inferred metabolic profiles of bacterial genomes**.

Table S3 **Detailed information for putative genes associated with key metabolic pathways of co-occurring bacteria in bioleaching heaps**.

Table S4 **Comparisons of predicted CAZymes in the genomes of ***L. ferriphilum, A. caldus***, and ***S. thermosulfidooxidans*****.
