# Supplementary figures and images for: Comparative Genomics Unravels the Functional Roles of Co-occurring Acidophilic Bacteria in Bioleaching Heaps
Source: Front Microbiol. 2017 May 5;8:790. doi: 10.3389/fmicb.2017.00790 (PMC5418355; doi:10.3389/fmicb.2017.00790)

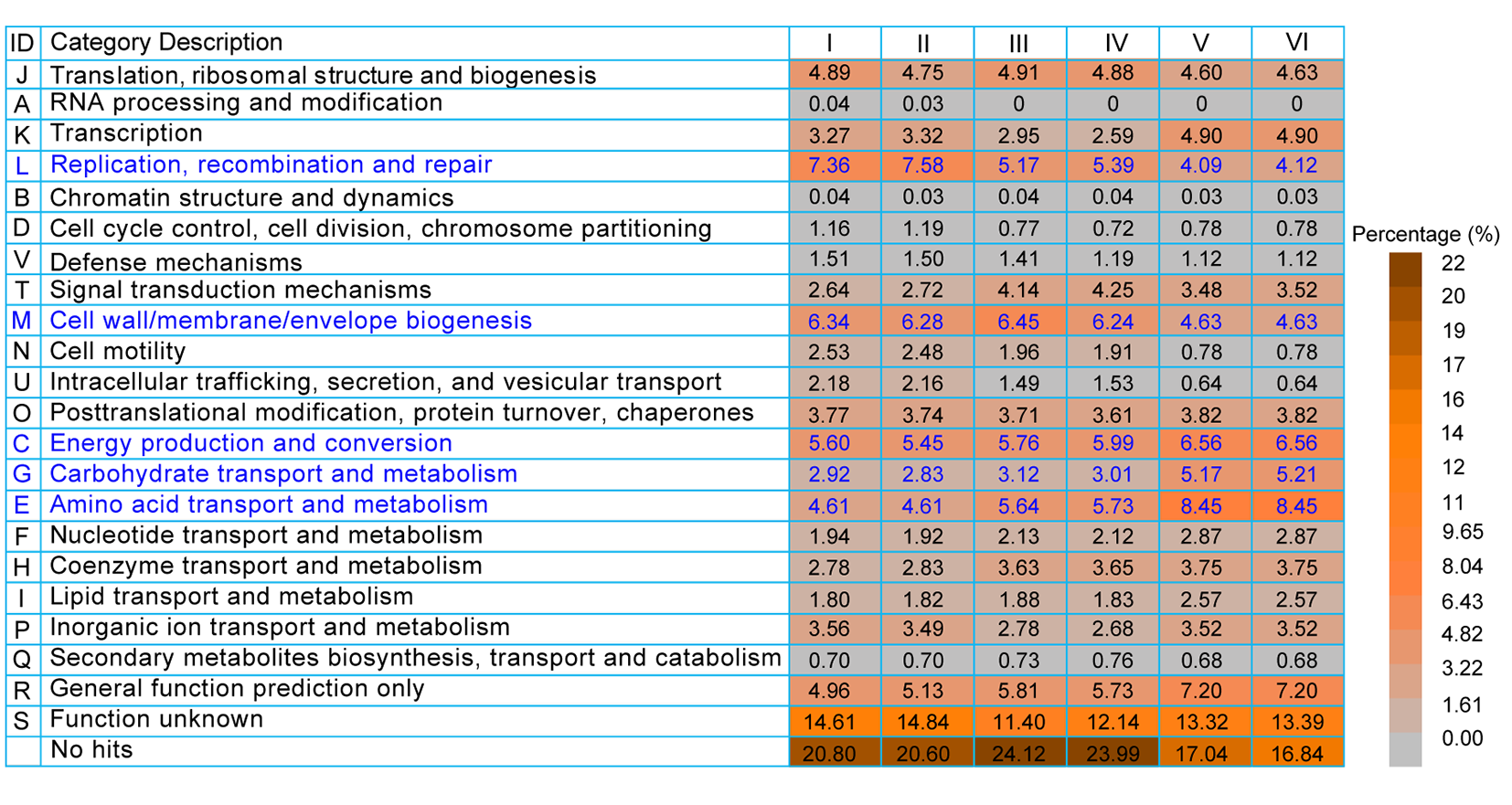

Supplement: Figure S1 — Heat map depicting the percentages of genes assigned to the COG classification in A. caldus DX (I), A. caldus ZJ (II), L. ferriphilum DX (III), L. ferriphilum ZJ (IV), S. thermosulfidooxidans DX (V), and S. thermosulfidooxidans ZJ (VI). The five abundant functional categories within all of the bacterial genomes are highlighted in blue. [file Image1.TIF]

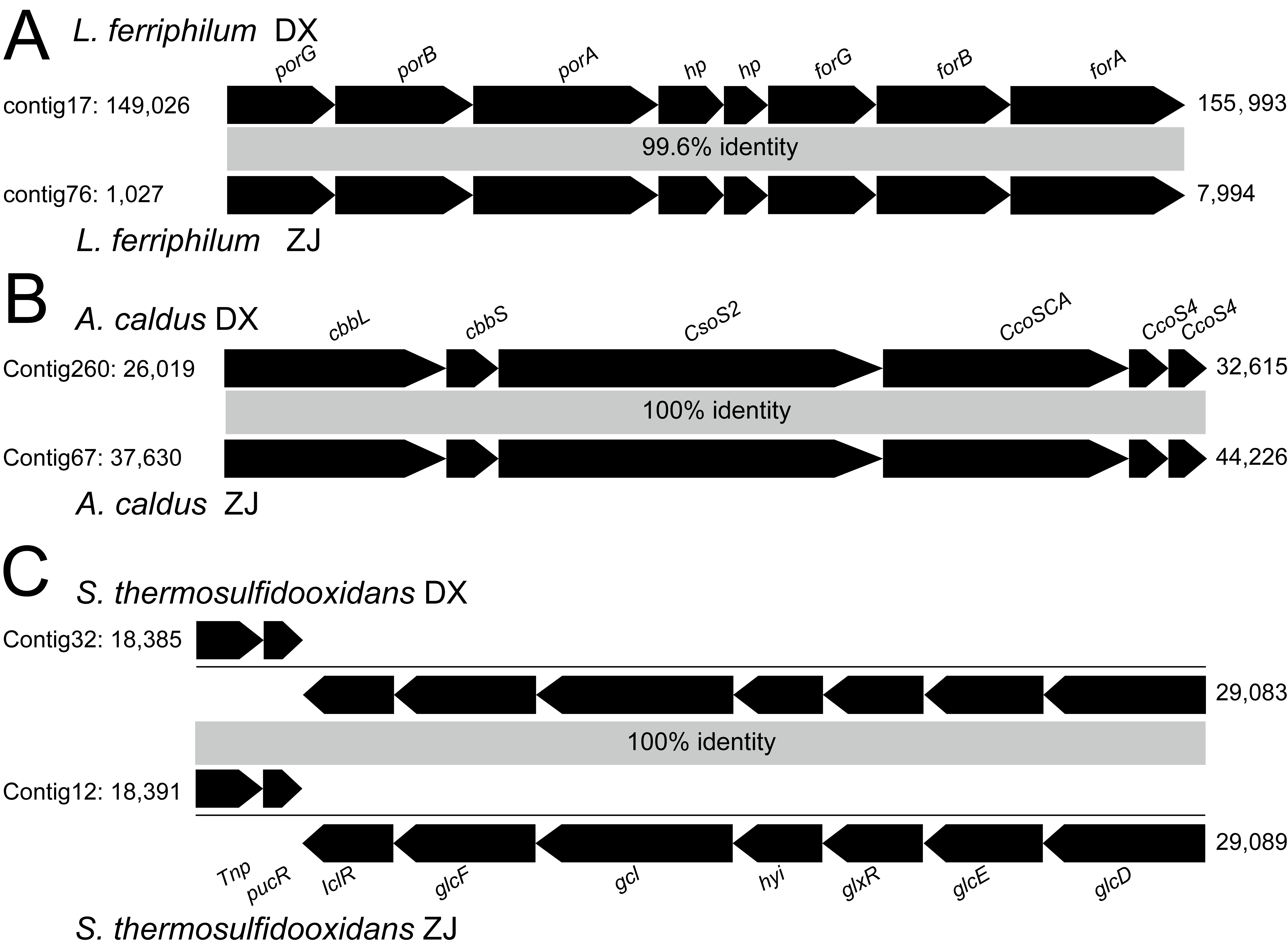

Supplement: Figure S2 — Homologous genome regions in individual strains associated with pyruvate ferredoxin oxidoreductase and 2-oxoglutarate ferredoxin oxidoreductase (A), carboxysome (B), and glycolate oxidase (C). porG, pyruvate ferredoxin oxidoreductase gamma subunit; porB, pyruvate ferredoxin oxidoreductase beta subunit; porA, pyruvate ferredoxin oxidoreductase alpha subunit; hp, hypothetical protein; forG, 2-oxoglutarate ferredoxin oxidoreductase gamma subunit; forB, 2-oxoglutarate ferredoxin oxidoreductase beta subunit; forA, 2-oxoglutarate ferredoxin oxidoreductase alpha subunit; cbbL, Rubisco large chain; cbbS, Rubisco small chain; CsoS2, carboxysome shell protein CsoS2; CsoSCA, carboxysome-associated carbonic anhydrase; CsoS4, carboxysome shell protein CsoS4; Tnp, transposase; pucR, PucR family transcriptional regulator; IclR, IclR family transcriptional regulator; glcF, glycolate oxidase iron-sulfur subunit; gcl, glyoxylate carboligase; hyi, hydroxypyruvate isomerase; glxR, 2-hydroxy-3-oxopropionate reductase; glcE, glycolate oxidase FAD binding subunit; glcD, glycolate oxidase subunit GlcD. [file Image2.TIF]

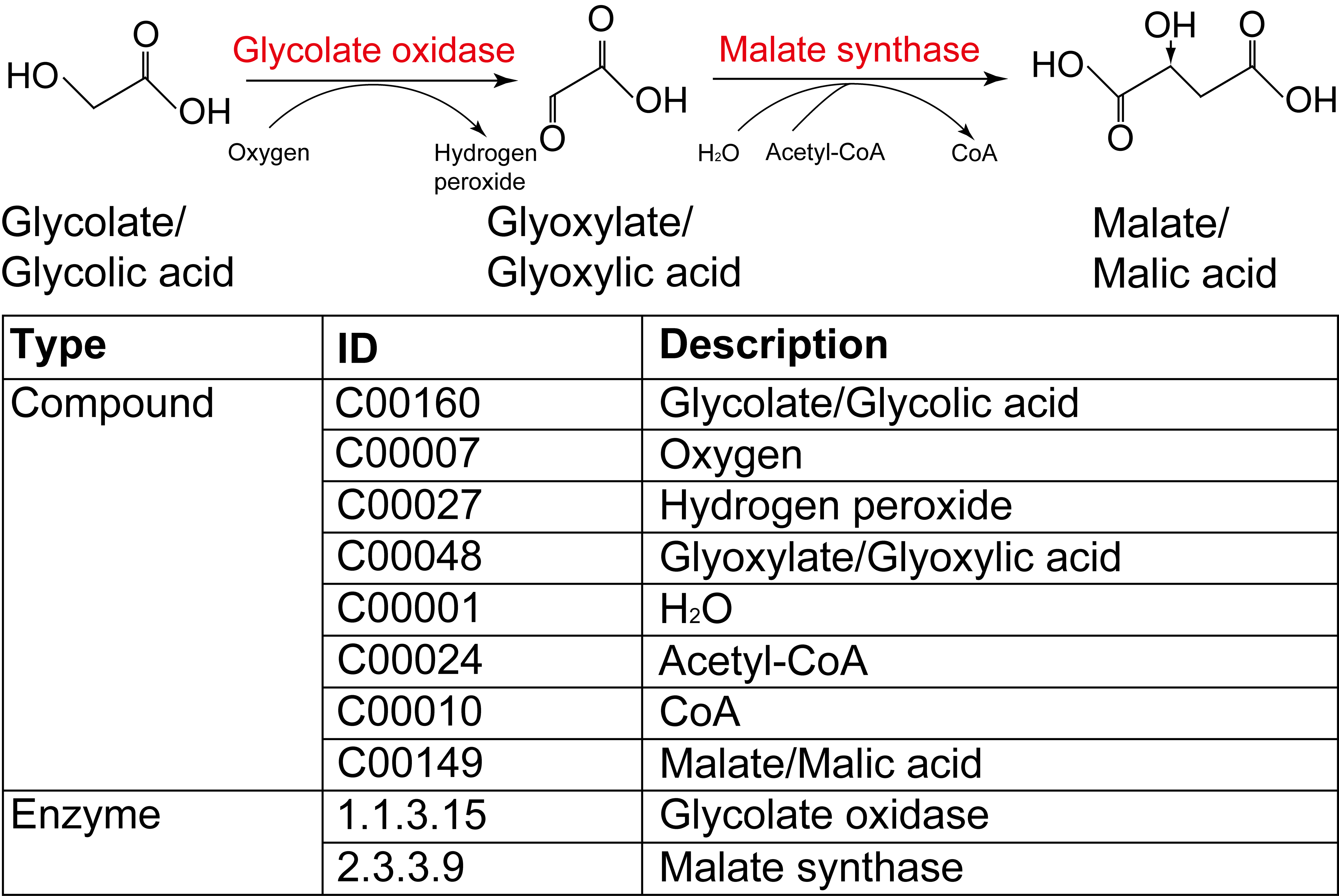

Supplement: Figure S3 — Putative route for glycolic acid assimilation within S. thermosulfidooxidans strains. [file Image3.TIF]
