# Supplementary material for: Comparative Genomics Unravels the Functional Roles of Co-occurring Acidophilic Bacteria in Bioleaching Heaps
Source: Front Microbiol. 2017 May 5;8:790. doi: 10.3389/fmicb.2017.00790 (PMC5418355; doi:10.3389/fmicb.2017.00790)
Supplement: Table S2 — Comparisons of the inferred metabolic profiles of bacterial genomes. [file Table2.DOCX]

**Table S2** Comparisons of the inferred metabolic profiles of bacterial genomes.

| **Pathway** | **Number of CDS** | | | | | |
| --- | --- | --- | --- | --- | --- | --- |
|  | ***Leptospirillum ferriphilum*** | | ***Acidithiobacillus caldus*** | | ***Sulfobacillus thermosulfidooxidans*** | |
|  | **DX** | **ZJ** | **DX** | **ZJ** | **DX** | **ZJ** |
| Carbohydrate metabolism | **209** | **200** | **198** | **200** | **256** | **257** |
| Energy metabolism | **113** | **116** | **140** | **138** | **168** | **168** |
| Lipid metabolism | 35 | 32 | 37 | 37 | 44 | 44 |
| Nucleotide metabolism | 76 | 76 | 90 | 89 | 99 | 99 |
| Amino acid metabolism | **144** | **141** | **156** | **159** | **244** | **244** |
| Metabolism of other amino acids | 25 | 24 | 29 | 29 | 53 | 53 |
| Glycan biosynthesis and metabolism | 40 | 40 | 44 | 43 | 24 | 24 |
| Metabolism of cofactors and vitamins | **109** | **110** | **109** | **110** | **139** | **139** |
| Metabolism of terpenoids and polyketides | 27 | 25 | 29 | 29 | 29 | 29 |
| Biosynthesis of other secondary metabolites | 26 | 26 | 36 | 36 | 36 | 36 |
| Xenobiotics biodegradation and metabolism | 28 | 28 | 14 | 15 | 32 | 32 |

The four most abundant metabolism-related genes are highlighted *in bold*.
