# Supplementary material for: Comparative Genomics Unravels the Functional Roles of Co-occurring Acidophilic Bacteria in Bioleaching Heaps
Source: Front Microbiol. 2017 May 5;8:790. doi: 10.3389/fmicb.2017.00790 (PMC5418355; doi:10.3389/fmicb.2017.00790)
Supplement: Table S4 — Comparisons of predicted CAZymes in the genomes of L. ferriphilum, A. caldus, and S. thermosulfidooxidans. [file Table4.DOCX]

**Table S4** Comparisons of predicted CAZymes in the genomes of *L. ferriphilum*, *A. caldus*, and *S. thermosulfidooxidans*.

| **Carbohydrate-active enzymes^*^** | **Number of CDS** | | | | | |
| --- | --- | --- | --- | --- | --- | --- |
|  | ***Leptospirillum ferriphilum*** | | ***Acidithiobacillus caldus*** | | ***Sulfobacillus thermosulfidooxidans*** | |
|  | **DX** | **ZJ** | **DX** | **ZJ** | **DX** | **ZJ** |
| **Glycoside Hydrolases (GHs)** | **23** | **24** | **26** | **26** | **45** | **46** |
| GH1 | 0 | 0 | 0 | 0 | 2 | 2 |
| GH3 | 1 | 1 | 1 | 1 | 1 | 1 |
| GH4 | 0 | 0 | 0 | 0 | 1 | 1 |
| GH8 | 1 | 1 | 1 | 1 | 2 | 2 |
| GH13 | 5 | 5 | 2 | 2 | 3 | 4 |
| GH15 | 2 | 2 | 2 | 2 | 4 | 4 |
| GH18 | 0 | 0 | 0 | 0 | 8 | 8 |
| GH19 | 0 | 0 | 1 | 1 | 0 | 0 |
| GH23 | 5 | 5 | 12 | 12 | 3 | 3 |
| GH31 | 0 | 0 | 0 | 0 | 3 | 3 |
| GH33 | 0 | 0 | 0 | 0 | 1 | 1 |
| GH35 | 0 | 0 | 0 | 0 | 1 | 1 |
| GH57 | 3 | 3 | 2 | 2 | 1 | 1 |
| GH73 | 0 | 0 | 2 | 2 | 0 | 0 |
| GH74 | 3 | 3 | 0 | 0 | 8 | 8 |
| GH76 | 0 | 0 | 0 | 0 | 1 | 1 |
| GH78 | 1 | 1 | 0 | 0 | 1 | 1 |
| GH84 | 0 | 0 | 0 | 0 | 1 | 1 |
| GH93 | 0 | 0 | 0 | 0 | 1 | 1 |
| GH103 | 0 | 0 | 1 | 1 | 0 | 0 |
| GH108 | 0 | 1 | 0 | 0 | 0 | 0 |
| GH109 | 1 | 1 | 1 | 1 | 2 | 2 |
| GH113 | 0 | 0 | 0 | 0 | 1 | 1 |
| GH114 | 0 | 0 | 1 | 1 | 0 | 0 |
| GH123 | 1 | 1 | 0 | 0 | 0 | 0 |
| **GlycosylTransferases (GTs)** | **46** | **46** | **42** | **42** | **58** | **58** |
| GT1 | 1 | 1 | 1 | 1 | 0 | 0 |
| GT2 | 12 | 13 | 13 | 13 | 15 | 15 |
| GT4 | 11 | 10 | 10 | 10 | 24 | 24 |
| GT5 | 1 | 1 | 1 | 1 | 0 | 0 |
| GT9 | 10 | 10 | 4 | 4 | 0 | 0 |
| GT19 | 1 | 1 | 1 | 1 | 1 | 1 |
| GT20 | 1 | 1 | 0 | 0 | 1 | 1 |
| GT21 | 1 | 1 | 2 | 2 | 0 | 0 |
| GT26 | 0 | 0 | 0 | 0 | 1 | 1 |
| GT27 | 0 | 0 | 0 | 0 | 1 | 1 |
| GT28 | 2 | 2 | 1 | 1 | 3 | 3 |
| GT30 | 1 | 1 | 2 | 2 | 0 | 0 |
| GT35 | 1 | 1 | 2 | 1 | 0 | 0 |
| GT39 | 0 | 0 | 0 | 0 | 2 | 2 |
| GT51 | 2 | 2 | 3 | 3 | 3 | 3 |
| GT66 | 0 | 0 | 0 | 0 | 1 | 1 |
| GT76 | 0 | 0 | 0 | 0 | 4 | 4 |
| GT81 | 0 | 0 | 0 | 0 | 1 | 1 |
| GT83 | 2 | 2 | 2 | 3 | 1 | 1 |
| **Polysaccharide Lyases (PLs)** | **0** | **0** | **1** | **1** | **1** | **1** |
| PL22 | 0 | 0 | 1 | 1 | 1 | 1 |
| **Carbohydrate Esterases (CEs)** | **6** | **6** | **7** | **6** | **33** | **33** |
| CE1 | 2 | 2 | 1 | 1 | 12 | 12 |
| CE4 | 3 | 3 | 1 | 1 | 7 | 7 |
| CE7 | 0 | 0 | 1 | 1 | 1 | 1 |
| CE9 | 0 | 0 | 0 | 0 | 2 | 2 |
| CE10 | 0 | 0 | 3 | 2 | 9 | 9 |
| CE11 | 1 | 1 | 1 | 1 | 0 | 0 |
| CE14 | 0 | 0 | 0 | 0 | 2 | 2 |
| **Auxiliary Activities (AAs)** | **0** | **0** | **3** | **2** | **9** | **9** |
| AA3 | 0 | 0 | 0 | 0 | 1 | 1 |
| AA4 | 0 | 0 | 0 | 0 | 3 | 3 |
| AA6 | 0 | 0 | 1 | 1 | 1 | 1 |
| AA7 | 0 | 0 | 2 | 1 | 4 | 4 |
| **Carbohydrate-Binding Modules (CBMs)** | **5** | **5** | **13** | **13** | **10** | **10** |
| CBM34 | 0 | 0 | 0 | 0 | 1 | 1 |
| CBM37 | 0 | 0 | 0 | 0 | 1 | 1 |
| CBM44 | 0 | 0 | 1 | 1 | 0 | 0 |
| CBM48 | 2 | 2 | 2 | 2 | 1 | 1 |
| CBM50 | 3 | 3 | 10 | 10 | 7 | 7 |
| **Total Number** | **80** | **81** | **92** | **90** | **156** | **157** |

**^*^** Family classification of the carbohydrate-active enzymes and their detailed description were shown in the CAZy database available at http://www.cazy.org/.
